# Supplementary material for: Visual analysis of geographical distribution of poets in Song China based on Complete Song Poetry
Source: PLoS One. 2024 Sep 6;19(9):e0310115. doi: 10.1371/journal.pone.0310115 (PMC11379205; doi:10.1371/journal.pone.0310115)
Supplement: S1 File — (PDF) [file pone.0310115.s003.pdf]

Visual analysis of geographical distribution of poets in Song China based on *Complete Song Poetry*

Enhai Lei[1, 2*], Xudong Hu[1, 2]

[1] School of Chinese Languages and Literatures, Lanzhou University, Lanzhou, P. R. China
[2] Center for Chinese Studies, Lanzhou University, Lanzhou, P. R. China

S1 Supporting Information for references, citations and figures

Contents

S1-1 Further statements to some references....................................... 2

S1-2 Citation in original Chinese.. 8

S1-3 English-Chinese translation of place names in figures.............. 10

S1-1 Further statements to some references

This study is a visual analysis of the geographical distribution of poets in Song China. In the process of analysis and discussion, it is inevitable to cite ancient Chinese literatures and published books. Therefore, we have made effort to provide further explanations of some of the references through webpage links here.

Reference

1. Zeng DX. *Zhongguo lidai wenxuejia zhi dilifenbu* (Geographical Distribution of Chinese Literary Masters of Previous Dynasties). Wuhan: Hubei jiaoyu chubanshe (Hubei Education Press); 1995.

Introduction of Prof. Zeng Daxing from Guangzhou University:
https://rw-gzhu-edu-cn.translate.goog/info/1146/5780.htm?_x_tr_sl=zh-CN&_x_tr_tl=en&_x_tr_hl=en&_x_tr_pto=sc

Zhongguo lidai wenxuejia zhi dilifenbu (Geographical Distribution of Chinese Literary Masters of Previous Dynasties)
中國歷代文學家之地理分佈
ISBN: 7-5351-1639-6
URL from Google Books:
https://books-google-com-hk.translate.goog/books?id=jW91AAAAIAAJ&q=%E4%B8%AD%E5%9B%BD%E5%8E%86%E4%BB%A3%E6%96%87%E5%AD%A6%E5%AE%B6%E5%9C%B0%E7%90%86%E5%88%86%E5%B8%83&dq=%E4%B8%AD%E5%9B%BD%E5%8E%86%E4%BB%A3%E6%96%87%E5%AD%A6%E5%AE%B6%E5%9C%B0%E7%90%86%E5%88%86%E5%B8%83&_x_tr_sl=zh-CN&_x_tr_tl=en&_x_tr_hl=en&_x_tr_pto=sc

URL from National Digital Library of China:
http://find.nlc.cn/search/showDocDetails?docId=4663648351138184523&dataSource=ucs01&query=%E4%B8%AD%E5%9C%8B%E6%AD%B7%E4%BB%A3%E6%96%87%E5%AD%B8%E5%AE%B6%E4%B9%8B%E5%9C%B0%E7%90%86%E5%88%86%E4%BD%88

Hubei jiaoyu chubanshe (Hubei Education Press)
Official website: http://www.hbedup.com/

2. Tan ZB. *Zhongguo wen xue jia da ci dian* (Dictionary of Chinese Literati). Shanghai: Shanghai shudian (Shanghai Bookstore Publishing House); 1981.

Introduction of Tan Zhengbi from Wikipidia:
https://zh-m-wikipedia-org.translate.goog/wiki/%E8%B0%AD%E6%AD%A3%E7%92%A7?_x_tr_sl=zh-

TW&_x_tr_tl=en&_x_tr_hl=en&_x_tr_pto=sc

Zhongguo wen xue jia da ci dian (Dictionary of Chinese Literati)
中國文學家大辭典
URL from Google Books:
https://books-google-com.translate.goog/books?id=mUWa0AEACAAJ&printsec=frontcover&dq=editions:LCCNc64002149&_x_tr_sl=zh-TW&_x_tr_tl=en&_x_tr_hl=en&_x_tr_pto=sc

Information for *Shanghai shudian* (Shanghai Bookstore Publishing House):
https://connect.ccbookfair.com/en/showroom-2023/institutions/0e28938

3. Wang ZP, Liu X. *Songci zuozhe tongji fenxi* (Statistical Analysis of Authors in Song Ci). Literature & Art Studies. 2003; 06: 54-59.

Introduction of Prof. Wang Zhaopeng from South-Central Minzu University:
https://www-scuec-edu-cn.translate.goog/literature/info/1010/1013.htm?_x_tr_sl=zh-CN&_x_tr_tl=en&_x_tr_hl=en&_x_tr_pto=sc

URL from CNKI to reach the paper:
https://www.cnki.net/KCMS/detail/detail.aspx?dbcode=CJFD&dbname=CJFD2003&filename=WYYJ200306008&uniplatform=OVERSEA&v=9jkq0Vn8LIl_ODzYyb3_DaKjTgz7QdAVYUijn8mOtgjDp7s-YiETbC6DgTefwsXf

4. Wang X. *Beisong Shiren de dilifenbu jiqi wenxuyeshi yiyifenxi* (The Gographical Distribution of Poets in the Northern Song Dynasty and Its Literary Significance). Literary Heritage. 2006; 06: 52-62.

Introduction of Prof. Wang Xiang from Baidu:
https://baike-baidu-com.translate.goog/item/%E7%8E%8B%E7%A5%A5/6060586?_x_tr_sl=zh-CN&_x_tr_tl=en&_x_tr_hl=en&_x_tr_pto=sc

URL from CNKI to reach the paper:
https://www.cnki.net/KCMS/detail/detail.aspx?dbcode=CJFD&dbname=CJFD2006&filename=WXYC200606006&uniplatform=OVERSEA&v=4a6jPx-GkLFX1CfM2sJBf4LnBZkKcNafYvjVhwvlh0-LhPWnvb-ZDKOMf-FF--Oz

5. Center for Ancient Chinese Classics and Archives of Peking University, ed. *Quansongshi*(Complete Song Poetry).Peking University Press. 1998.

Complete Song Poetry (Quansongshi 全宋詩)
Further information for *Complete Song Poetry* from Chinaknowledge.de:
http://www.chinaknowledge.de/Literature/Poetry/quansongshi.html

Peking University Press:
Official website:
https://www.pup.cn

6. Lou XX. *Quansongci zuozhe de dilifenbu jiqi chengyin* (Geographical Distribution of Authors in *Complete Song Iambic Verse Collection* and the Cause). Journal of Taizhou University. 2020; 42(02): 57-62.
https://link.oversea.cnki.net/doi/10.13853/j.cnki.issn.1672-3708.2020.02.012

Introduction of Dr. Lou Xinxing from Taizhou University:
https://rw.tzc.edu.cn/info/1009/2945.htm

7. Department of Chinese Language and Literature of Peking University; 2005[cited 4 Feb 2024] Complete Song Poetry Analysis System.
http://www.chinabooktrading.com/song

This system can be accessed through the website of the National Digital Library of China. A free account registration is required:
http://read.nlc.cn/outRes/outResList?type=%E5%85%A8%E9%83%A8&searchName=%E5%85%A8%E5%AE%8B%E8%AF%97%E5%88%86%E6%9E%90%E7%B3%BB%E7%BB%9F

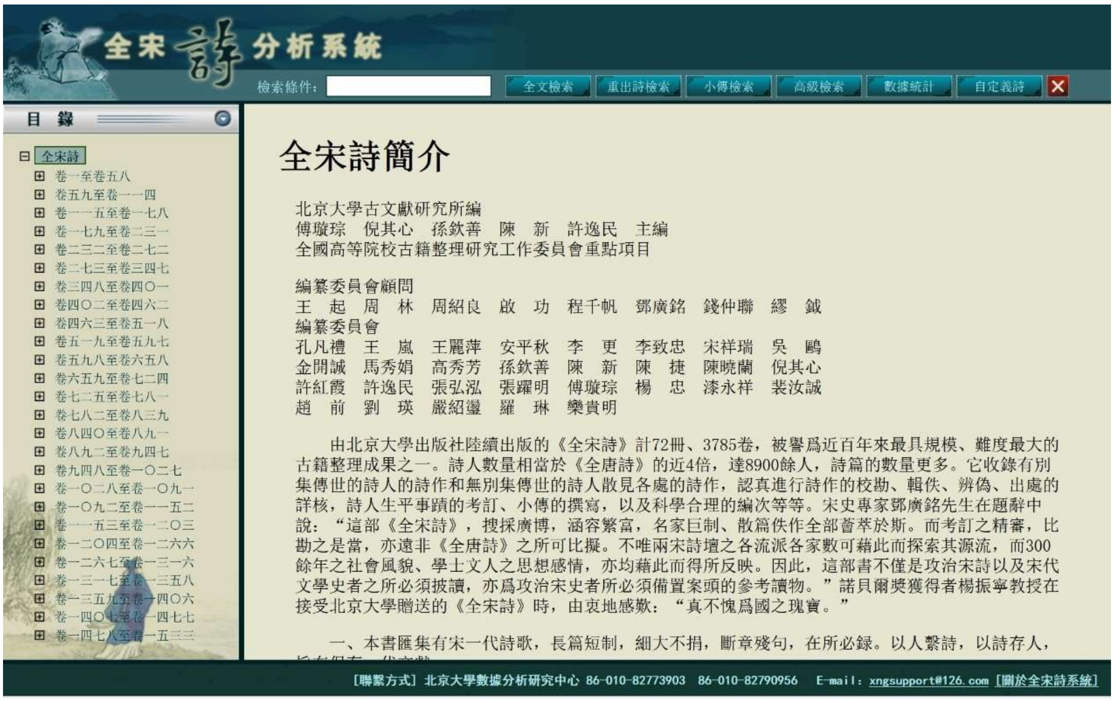

A screenshot of Complete Song Poetry Analysis System.

14. Li MY. *Chongxiu Nanhai Xianzhi Xu* (Preface to the Revised Gazetteer of Nanhai County). In Dai ZC, Shi C, eds. *Guangzhoufuzhi* (Gazetteer of Guangzhou Prefecture), Guangzhou: Yuexiu Shuyuan. 1879; vol.15.http://read.nlc.cn/OutOpenBook/OpenObjectBook?aid=403&bid=81365.0
This URL link is from National Digital Library of China (https://www.nlc.cn).

15. Wang ZX. *Qianxueji* (Relocation Record of School) In Dai ZC, Shi C, eds. *Guangzhoufuzhi* (Gazetteer of Guangzhou Prefecture), Guangzhou: Yuexiu Shuyuan. 1879; vol.15.

http://read.nlc.cn/OutOpenBook/OpenObjectBook?aid=403&bid=81365.0
This URL link is from National Digital Library of China (https://www.nlc.cn).

16. Li B. *Sikong Hou Andu Miaoji* (Memorial Temple of Hou Andu, the Minister of Public Works). In Dong G, Ruan Y, Xu S, et al. *Quan tang wen* (Complete Works of Tang Dynasty). Beijing: Zhonghua shuju (Zhonghua Book Company). 1983; vol. 712, p. 7311.

Li Bo (773-831)
Introduction of Li Bo from Wikipedia:
https://zh-m-wikipedia-org.translate.goog/wiki/%E6%9D%8E%E6%B8%A4?_x_tr_sl=zh-TW&_x_tr_tl=en&_x_tr_hl=en&_x_tr_pto=sc

Quan tang wen 全唐文
Further information for Quan tang wen from Chinaknowledge.de:
http://www.chinaknowledge.de/Literature/Poetry/quantangwen.html

Zhonghua shuju (Zhonghua Book Company):
Introduction from Wikipedia:
https://en.wikipedia.org/wiki/Zhonghua_Book_Company
Official website:
http://www.zhbc.com.cn/zhsj/fg/home/home.html

17. Su S. *Chaozhou Han Wengong Miao Bei* (Inscription on the Temple Tablet of Han Wen Gong in Chaozhou). In Kong, FL, ed, *Sushiwenji* (Collected Works of Su Shi). Beijing: Zhonghua Shuju (Zhonghua Book Company). 1986; p509.

Su Shi (1037-1101)
Introduction of Su Shi from Wikipedia:
https://en.wikipedia.org/wiki/Su_Shi

Sushiwenji (Collected Works of Su Shi) 蘇軾文集
ISBN: 9787101006742
URL from Google Books:
https://books-google-com.translate.goog/books/about/%E8%98%87%E8%BB%BE%E6%96%87%E9%9B%86.html?id=ZFxdnQEACAAJ&_x_tr_sl=zh-TW&_x_tr_tl=en&_x_tr_hl=en&_x_tr_pto=sc

14. Li SC. Shuyuanji (Records of the Academy). In Zhou SX, ed, *Chaozhoufuzhi* (Gazetteer of Chaozhou Prefecture). Chaozhou: Baoanzongju. 1893; vol 12.
http://read.nlc.cn/OutOpenBook/OpenTwoObjectBook?aid=403&bid=105381.0&cid=157056
This URL link is from National Digital Library of China (https://www.nlc.cn).

16. Zhang J. *Qiandao Siming Tujing* (Siming Map and Classics in Qiandao regin), vol.

1. In *Songyuan fangzhi congkan* (Collected Works on Song and Yuan Local Chronicles) Beijing: Zhonghua Shuju (Zhonghua Book Company). 1990; p. 4877.

Information for Zhang Jin and Qiandao Siming Tujing from Baidu:
https://baike-baidu-com.translate.goog/item/%E4%B9%BE%E9%81%93%E5%9B%9B%E6%98%8E%E5%9B%BE%E7%BB%8F/9138642?_x_tr_sl=zh-CN&_x_tr_tl=en&_x_tr_hl=en&_x_tr_pto=sc

Songyuan fangzhi congkan (Collected Works on Song and Yuan Local Chronicles)
宋元方志叢刊
ISBN: 978-7101005691
URL from Google Books:
https://books-google-com.translate.goog/books/about/%E5%AE%8B%E5%85%83%E6%96%B9%E5%BF%97%E5%8F%A2%E5%88%8A.html?id=ujnTAAAAMAAJ&_x_tr_sl=zh-TW&_x_tr_tl=en&_x_tr_hl=en&_x_tr_pto=sc

20. Li T. *Xu Zizhitongjian Changbia* (Continuation of Comprehensive Mirror for Aid in Government: Extended Compilation). Beijing: Zhonghua Shuju (Zhonghua Book Company). 2004; vol. 118, p. 2783.
Continuation of Comprehensive Mirror for Aid in Government: Extended Compilation (*Xu Zizhitongjian Changbia* 續資治通鑑長編)

Further information from chinaknowledge.de:
http://www.chinaknowledge.de/Literature/Historiography/xuzizhitongjianchangbian.html

URL from Wikipidia:
https://en.wikipedia.org/wiki/Xu_Zizhi_Tongjian_Changbian

URL from Google Books:
https://books-google-com.translate.goog/books?id=wG49tQEACAAJ&dq=%E7%BB%AD%E8%B5%84%E6%B2%BB%E9%80%9A%E9%89%B4%E9%95%BF%E7%BC%96&_x_tr_sl=zh-CN&_x_tr_tl=en&_x_tr_hl=en&_x_tr_pto=sc

21. Li GF. *Luoyangmingyuanji* (Famous Gardens in Luoyang). In Chen, Z, Zhang GC, eds. *Zhongguo Lidai Mingyuanji Xuanzhu* (Selected notes of Famous Gadens in China). *Hefei: Anhui kexuejishu chubanshe*. 1983; p. 38

Introduction of Li Gefei and Gardens in Luoyang from Wikipedia:
https://zh-m-wikipedia-org.translate.goog/wiki/%E6%9D%8E%E6%A0%BC%E9%9D%9E?_x_tr_sl=zh-TW&_x_tr_tl=en&_x_tr_hl=en&_x_tr_pto=sc

URL from Google Books:
https://books-google-com.translate.goog/books/about/%E4%B8%AD%E5%9B%BD%E5%8E%86%E4%BB%A3%E5%90%8D%E5%9B%AD%E8%AE%B0%E9%80%89%E6%B3%A8.html?id=us3SSgAACAAJ&_x_tr_sl=zh-CN&_x_tr_tl=en&_x_tr_hl=en&_x_tr_pto=sc

22. Zhu SS. *Lun Songdaiwenhua Zhong de "Meishan Xianxiang"* (On the "Meishan Phenomenon" in Song Culture). Journal of Sichuan University (Social Science Edition), 2004; 03: 105-110.

Introduction of Prof.Zhu Shangshu from Baidu
https://baike-baidu-com.translate.goog/item/%E7%A5%9D%E5%B0%9A%E4%B9%A6/11033992?_x_tr_sl=zh-CN&_x_tr_tl=en&_x_tr_hl=en&_x_tr_pto=sc

URL from CNKI to reach the paper:
https://www.cnki.net/KCMS/detail/detail.aspx?dbcode=CJFD&dbname=CJFD2004&filename=SCDZ200403020&uniplatform=OVERSEA&v=iTWRkyxmT5d9bFRoSMtGjnlckRaXleYcf6ZKUUatraQ2AmWVC9z1clQ004h9lH7H

S1-2 Citation in original Chinese

Some of the citations come from ancient Chinese works, and the original text is shown below:

1. "Guangzhou has always been known by its abundance in natural resources... massive bananas and nuts are grown here, a large amount of shrimps and crabs are raised here. Meanwhile, Schools and temples are scattered here. Locals are willing to have good teachers to educate their children. The sound of strings and reciting poems complement each other. Although the number of talented people participating in the imperial examination is less than that in the Central China, the number of people passing it is the same as in the Central China." [14]

Original text:

"廣素號富饒……雖蕉阜桄林之墟，蠣田蠏窟之嶼，皆渠渠齋廬，幣良師以玉其子弟，弦歌琤相聞，挾藝待試上都者，數甚嗇，每連聯登名與中州等。"

2. "Dongguan was initially a county and later transformed into a center for supervising county governance, a model role that persists to this day. The land here is fertile, and the number of talented people with sophisticated farming skills, war skills, and famous official positions surpassed other places." [15]

Original text:

"東莞, 故郡也。後為監郡。監榜猶在。及為縣。地軼賦夥, 長材秀民之戰藝有司者, 倍他邑。"

3. "Qujiang in Shaozhou is the hometown of generals and ministers." [16]

Original text:

"韶之曲江, 所謂將相之鄉也。"

4. "At first, the people of Chaozhou were not very knowledgeable, so Han Yu appointed a local scholar Zhao De to teach them. Since then, people in Chaozhou have attached great importance to cultural cultivation, and this trend has also spread to ordinary people, which has continued to this day and is known as good governance." [17]

Original text:

"始潮人未知學，公命進士趙德為之師。自是潮之士，皆篤于文行，延及齊民，至於今，號稱易治。"

5. "Before the Tang Dynasty, Chaozhou's reputation and educational level were not very high, and its cultural heritage was not rich enough. However, the beautiful natural scenery and magical atmosphere here are hidden in dense forests, wetlands, and cities. Since Changli, courtesy name of Han Yu, became the governor of Chaozhou and Zhao De became his mentor, scholars here began to realize the importance of learning. As a result, the landscape of Chaozhou has been revitalized." [18]

Original text:

“潮州自唐以前，声教罕通，文物未着，山川灵异之气，半湮於荆榛瘴疠山林海市之中，自昌黎出守，赵德为师，士始知学，山川之色，亦遂烂然一新。”

6. “Mingzhou is in the eastern part of Yue. If you look at the map, it is in a rather remote corner. Although not a metropolis, it is an important hub for maritime transportation. It is bounded by Fujian and Guangzhou in the south, faces Japan in the east, and is neighbored by Koera in the north. Numerous merchant ships pass by, making it a flourishing commercial hub. Heading east to Dinghai, you will encounter Jiaomen and Hudun mountain, making Mingzhou an important rendezvous point in the southeast as well.” [19]

Original text:

“明之為州，實越之東部，觀輿地圖，則僻在一隅。雖非都會，乃海道輻湊之地，故南則閩廣，東則倭人，北則高勾麗，商舶往來，物貨豐衍。東出定海，有蛟門虎蹲天設之險。亦東南之要會也。”

7. “During times of peace, one can reside in the bustling and well-connected Kaifeng as it benefits from its convenient transportation. However, during war times, Luoyang's formidable mountains and rivers offer a stronghold to protect the Central China.” [20]

Original text:

“太平則居東京通濟之地，以便天下;急難則居西洛險固之宅，以守中原。”

S1-3 English-Chinese translation of place names in figures

Chaohu, Anhui	安徽巢湖
Fengyang, Anhui	安徽鳳陽
Hanshan, Anhui	安徽含山
Langxi, Anhui	安徽郎溪
Susong, Anhui	安徽宿松
Suzhou, Anhui	安徽宿州
Wuhu, Anhui	安徽蕪湖
Yongcheng, Anhui	安徽永城
Changtai, Fujian	福建長泰
Dehua, Fujian	福建德化
Futang (Fuqing) , Fujian	福建福唐（福清）
Longxi, Fujian	福建龍溪
Pingnan, Fujian	福建屏南
Xiamen, Fujian	福建厦門
Shouning, Fujian	福建壽寧
Songxi, Fujian	福建松溪
Wuping, Fujian	福建武平
Yunxiao, Fujian	福建雲霄
Jingning, Gansu	甘肅靜寧
Minxian, Gansu	甘肅岷縣
Ningxian, Gansu	甘肅寧縣
Wenxian, Gansu	甘肅文縣
Xihe, Gansu	甘肅西和
Bobai, Guangxi	廣西博白
Chao'an, Guangdong	廣東潮安
Guishan, Guangdong	廣東歸善
Heyuan, Guangdong	廣東河源
Huilai, Guangdong	廣東惠來
Jieyang, Guangdong	廣東揭陽
Longchuan, Guangdong	廣東龍川
Nanhai, Guangdong	廣東南海
Shaoguan, Guangdong	廣東曲江
Shenzhen, Guangdong	廣東深圳
Suixi, Guangdong	廣東遂溪
Xinxing, Guangdong	廣東新興
Xingning, Guangdong	廣東興寧
Yangjiang, Guangdong	廣東陽江
Zhongshan, Guangdong	廣東中山
Guixian, Guangxi	廣西貴縣

Hepu, Guangxi	廣西合浦
Hexian, Guangxi	廣西賀縣
Lingui, Guangxi	廣西臨桂
Lingshan, Guangxi	廣西靈山
Nanning, Guangxi	廣西南寧
Rongxian, Guangxi	廣西容縣
Rongshui, Guangxi	廣西融水
Tengxian, Guangxi	廣西藤縣
Tiandong, Guangxi	廣西田東
Yangshuo, Guangxi	廣西陽朔
Sanya, Hainan	海南三亞
Anxin, Hebei	河北安新
Baoding, Hebei	河北保定
Cangzhou, Hebei	河北滄州
Cheng'an, Hebei	河北成安
Fucheng, Hebei	河北阜城
Guangping, Hebei	河北廣平
Huailai, Hebei	河北懷來
Langfang, Hebei	河北廊坊
Lulong, Hebei	河北盧龍
Ningjin, Hebei	河北寧晉
Quyang, Hebei	河北曲陽
Quzhou, Hebei	河北曲周
Shahe, Hebei	河北沙河
Shenxian, Hebei	河北深縣
Yongnian, Hebei	河北永年
Yuanshi, Hebei	河北元氏
Baofeng, Henan	河南寶豐
Dengzhou, Henan	河南鄧州
Fengqiu, Henan	河南封丘
Jixian, Henan	河南汲縣
Xunxian, Henan	河南浚縣
Lankao, Henan	河南蘭考
Lingbao, Henan	河南靈寶
Luyi, Henan	河南鹿邑
Mengjin, Henan	河南孟津
Nanyang, Henan	河南南陽
Ningling, Henan	河南寧陵
Qixian, Henan	河南淇縣
Ruzhou, Henan	河南汝州
Taikang, Henan	河南太康
Tanghe, Henan	河南唐河

Tongxu, Henan	河南通許
Weishi, Henan	河南尉氏
Xihua, Henan	河南西華
Xin'an, Henan	河南新安
Xinxiang, Henan	河南新鄉
Yanling, Henan	河南鄢陵
Yancheng, Henan	河南郾城
Ezhou, Hubei	湖北鄂州
Gucheng, Hubei	湖北穀城
Guangshui, Hubei	湖北廣水
Huangmei, Hubei	湖北黃梅
Laohekou, Hubei	湖北老河口
Qianjiang, Hubei	湖北潛江
Tongcheng, Hubei	湖北通城
Yunmeng, Hubei	湖北雲夢
Baojing, Hunan	湖南保靖
Chaling, Hunan	湖南茶陵
Chenxian, Hunan	湖南郴縣
Guiyang, Hunan	湖南桂陽
Jiangyong, Hunan	湖南江永
Liling, Hunan	湖南醴陵
Longhui, Hunan	湖南隆回
Shaoyang, Hunan	湖南邵陽
Xiangxiang, Hunan	湖南湘鄉
Xinhua, Hunan	湖南新化
Yizhang, Hunan	湖南宜章
Yiyang, Hunan	湖南益陽
Yuanjiang, Hunan	湖南沅江
Yuanling, Hunan	湖南沅陵
Huzhou, Jiangsu	江蘇湖州
Huaiyin, Jiangsu	江蘇淮陰
Taicang, Jiangsu	江蘇太倉
Taixing, Jiangsu	江蘇泰興
Tongshan, Jiangsu	江蘇銅山
Yancheng, Jiangsu	江蘇鹽城
Fenyi, Jiangxi	江西分宜
Guangchang, Jiangxi	江西廣昌
Guangfeng, Jiangxi	江西廣豐
Jing'an, Jiangxi	江西靖安
Lichuan, Jiangxi	江西黎川
Nankang, Jiangxi	江西南康
Pengze, Jiangxi	江西彭澤

Qingjiang, Jiangxi	江西清江
Shangyou, Jiangxi	江西上猶
Taihe, Jiangxi	江西太和
Yuanzhou, Jiangxi	江西袁州
Guyuan, Ningxia	寧夏固原
Longde, Ningxia	寧夏隆德
Japan	日本
Anqiu, Shandong	山東安丘
Binxian, Shandong	山東濱縣
Binzhou, Shandong	山東濱州
Dingtao, Shandong	山東定陶
Feixian, Shandong	山東費縣
Gaomi, Shandong	山東高密
Heze, Shandong	山東菏澤
Huangxian, Shandong	山東黃縣
Huimin, Shandong	山東惠民
Jizhou, Shandong	山東濟州
Licheng, Shandong	山東歷城
Liaocheng, Shandong	山東聊城
Linqing, Shandong	山東臨清
Lingxian, Shandong	山東陵縣
Renping, Shandong	山東茌平
Shouguang, Shandong	山東壽光
Tengxian, Shandong	山東滕縣
Xiajin, Shandong	山東夏津
Yanggu, Shandong	山東陽穀
Leling, Shandong	山東樂陵
Zaozhuang, Shandong	山東棗莊
Zhangqiu, Shandong	山東章丘
Ziyang, Shandong	山東滋陽
Zouping, Shandong	山東鄒平
Gaoping, Shanxi	山西高平
Guangling, Shanxi	山西廣靈
Hongtong, Shanxi	山西洪洞
Jishan, Shanxi	山西稷山
Jiangxian, Shanxi	山西絳縣
Jiexiu, Shanxi	山西介休
Jincheng, Shanxi	山西晉城
Kelan, Shanxi	山西岢嵐
Linfen, Shanxi	山西臨汾
Lingchuan, Shanxi	山西陵川
Qingyuan, Shanxi	山西清源

Xixian, Shanxi	山西隰縣
Xinjiang, Shanxi	山西新絳
Yangcheng, Shanxi	山西陽城
Yonghe, Shanxi	山西永和
Yuxian, Shanxi	山西盂縣
Yuanping, Shanxi	山西原平
Zuoquan, Shanxi	山西左權
Chang'an, Shaanxi	陝西長安
Huxian, Shaanxi	陝西戶縣
Huayin, Shaanxi	陝西華陰
Huangling, Shaanxi	陝西黄陵
Ningqiang, Shaanxi	陝西寧强
Weinan, Shaanxi	陝西渭南
Wugong, Shaanxi	陝西武功
Xingping, Shaanxi	陝西興平
Xunyi, Shaanxi	陝西旬邑
Zhouzhi, Shaanxi	陝西周至
Baoshan, Shanghai	上海寶山
Anxian, Sichuan	四川安縣
Cangxi, Sichuan	四川蒼溪
Dazhou, Sichuan	四川達州
Emei, Sichuan	四川峨眉
Fengjie, Sichuan	四川奉節
Hongya, Sichuan	四川洪雅
Jiajiang, Sichuan	四川夾江
Jianwei, Sichuan	四川犍為
Luzhou, Sichuan	四川瀘州
Mingshan (Yaan), Sichuan	四川名山（雅安）
Nanxi, Sichuan	四川南溪
Pengzhou, Sichuan	四川彭州
Quxian, Sichuan	四川渠縣
Wenjiang, Sichuan	四川溫江
Yaan, Sichuan	四川雅安
Yanting, Sichuan	四川鹽亭
Yibin, Sichuan	四川宜賓
Lezhi, Sichuan	四川樂至
Zhongxian, Sichuan	四川忠縣
Jinxi, Zhejiang	浙江金溪
Pinghu, Zhejiang	浙江平湖
Taishun, Zhejiang	浙江泰順
Xiaoshan, Zhejiang	浙江蕭山
Xincheng, Zhejiang	浙江新城

Yujiang, Zhejiang	浙江餘江
Qijiang, Chongqing	重慶綦江
Jingde, Anhui	安徽旌德
Liuan, Anhui	安徽六安
Lujiang, Anhui	安徽廬江
Suzhou, Anhui	安徽宿縣
Taihe, Anhui	安徽太和
Taiping, Anhui	安徽太平
Beijing	北京
Anxi, Fujian	福建安溪
Ninghua, Fujian	福建寧化
Qingliu, Fujian	福建清流
Yongfu, Fujian	福建永福
Zhaoan, Fujian	福建詔安
Longxi, Gansu	甘肅隴西
Deqing, Guangdong	廣東德慶
Huizhou, Guangdong	廣東惠州
Lianshan, Guangdong	廣東連山
Shunde, Guangdong	廣東順德
Lechang, Guangdong	廣東樂昌
Quanzhou, Guangxi	廣西全州
Qiongshan, Hainan	海南瓊山
Anci, Hebei	河北安次
Cixian, Hebei	河北磁縣
Feixiang, Hebei	河北肥鄉
Huolu, Hebei	河北獲鹿
Lixian, Hebei	河北蠡縣
Nanpi, Hebei	河北南皮
Xingtai, Hebei	河北邢臺
Dengxian, Henan	河南鄧縣
Mixian, Henan	河南密縣
Neihuang, Henan	河南內黃
Xinzheng, Henan	河南新鄭
Yanjin, Henan	河南延津
Yiyang, Henan	河南宜陽
Yuanyang, Henan	河南原陽
Gongan, Hubei	湖北公安
Jiayu, Hubei	湖北嘉魚
Jingmen, Hubei	湖北荊門
Zaoyang, Hubei	湖北棗陽
Anren, Hunan	湖南安仁
Liuyang, Hunan	湖南瀏陽

Ningxiang, Hunan	湖南寧鄉
Taoyuan, Hunan	湖南桃源
Yueyang, Hunan	湖南岳陽
Yizheng, Jiangsu	江蘇儀徵
Ganxian, Jiangxi	江西贛縣
Hukou, Jiangxi	江西湖口
Wanan, Jiangxi	江西萬安
Xinjian, Jiangxi	江西新建
Yudu, Jiangxi	江西于都
Jining, Shandong	山東濟寧
Jinxiang, Shandong	山東金鄉
Penglai, Shandong	山東蓬萊
Wenshang, Shandong	山東汶上
Wudi, Shandong	山東無棣
Yanzhou, Shandong	山東兗州
Changzhi, Shanxi	山西長治
Qixian, Shanxi	山西祁縣
Wanrong, Shanxi	山西萬榮
Xiaoyi, Shanxi	山西孝義
Zezhou, Shanxi	山西澤州
Binxian, Shaanxi	陝西彬縣
Fengxiang, Shaanxi	陝西鳳翔
Heyang, Shaanxi	陝西合陽
Yangxian, Shaanxi	陝西洋縣
Jiading, Shanghai	上海嘉定
Fuling, Sichuan	四川涪陵
Jintang, Sichuan	四川金堂
Lixian, Sichuan	四川理縣
Neijiang, Sichuan	四川內江
Pengxian, Sichuan	四川彭縣
Rongxian, Sichuan	四川榮縣
Xichong, Sichuan	四川西充
Xinjin, Sichuan	四川新津
Xuanhan, Sichuan	四川宣漢
Yingshan, Sichuan	四川營山
Ziyang, Sichuan	四川資陽
Longyou, Zhejiang	浙江龍游
Qiantang, Zhejiang	浙江錢塘
Yuquan, Zhejiang	浙江於潛
Guichi, Anhui	安徽貴池
Chong'an, Fujian	福建崇安
Taining, Fujian	福建泰寧

Zhangpu, Fujian	福建漳浦
Korea	高麗
Boluo, Guangdong	廣東博羅
Meixian, Guangdong	廣東梅縣
Zengcheng, Guangdong	廣東增城
Gongcheng, Guangxi	廣西恭城
Zhongshan, Guangxi	廣西鍾山
Dongguang, Hebei	河北東光
Hejian, Hebei	河北河間
Jixian, Hebei	河北冀縣
Raoyang, Hebei	河北饒陽
Huaiyang, Henan	河南淮陽
Jiyuan, Henan	河南濟源
Tangyin, Henan	河南湯陰
Weihui, Henan	河南衛輝
Yanshi, Henan	河南偃師
Xingyang, Henan	河南滎陽
Yuxian, Henan	河南禹縣
Huangzhou, Hubei	湖北黃州
Suizhou, Hubei	湖北隨州
Chenzhou, Hunan	湖南郴州
Leiyang, Hunan	湖南耒陽
Xiangtan, Hunan	湖南湘潭
Youxian, Hunan	湖南攸縣
Xuyi, Jiangsu	江蘇盱眙
Yizheng, Jiangsu	江蘇儀征
Dayu, Jiangxi	江西大余
Jinxi, Jiangxi	江西金溪
Longnan, Jiangxi	江西龍南
Wannian, Jiangxi	江西萬年
Xingguo, Jiangxi	江西興國
Yujiang, Jiangxi	江西餘江
Chengwu, Shandong	山東成武
Qufu, Shandong	山東曲阜
Tai'an, Shandong	山東泰安
Zhucheng, Shandong	山東諸城
Datong, Shanxi	山西大同
Qingxu, Shanxi	山西清徐
Dali, Shaanxi	陝西大荔
Hanzhong, Shaanxi	陝西漢中
Yan'an, Shaanxi	陝西延安
Changning, Sichuan	四川長寧

Deyang, Sichuan	四川德陽
Pengshan, Sichuan	四川彭山
Pengxi, Sichuan	四川蓬溪
Shehong, Sichuan	四川射洪
Yilong, Sichuan	四川儀隴
Wuyi, Zhejiang	浙江武義
Mengcheng, Anhui	安徽蒙城
Nanling, Anhui	安徽南陵
Qimen, Anhui	安徽祁門
Shucheng, Anhui	安徽舒城
Jiangle, Fujian	福建將樂
Dunhuang, Gansu	甘肅敦煌
Qingyang, Gansu	甘肅慶陽
Weixian, Hebei	河北威縣
Linying, Henan	河南臨潁
Shangcai, Henan	河南上蔡
Wuchang, Hubei	湖北武昌
Wuhan, Hubei	湖北武漢
Ningyuan, Hunan	湖南寧遠
Jiangning, Jiangsu	江蘇江寧
Suichuan, Jiangxi	江西遂川
Xingzi, Jiangxi	江西星子
Yiyang, Jiangxi	江西弋陽
Lean, Jiangxi	江西樂安
Danxian, Shandong	山東單縣
Linyi, Shandong	山東臨沂
Weifang, Shandong	山東濰坊
Lantian, Shaanxi	陝西藍田
Jiange, Sichuan	四川劍閣
Suining, Sichuan	四川遂寧
Tongliang, Sichuan	四川銅梁
Xindu, Sichuan	四川新都
Zitong, Sichuan	四川梓潼
Jixian, Tianjin	天津薊縣
Songyang, Zhejiang	浙江松陽
Yongjia, Zhejiang	浙江永嘉
Zhuji, Zhejiang	浙江諸暨
Fanchang, Anhui	安徽繁昌
Qingyang, Anhui	安徽青陽
Lianjiang, Fujian	福建連江
Shunchang, Fujian	福建順昌
Zhenghe, Fujian	福建政和

Chaoyang, Guangdong	廣東潮陽
Chaozhou, Guangdong	廣東潮州
Nanxiong, Guangdong	廣東南雄
Guilin, Guangxi	廣西桂林
Zhuozhou, Hebei	河北涿州
Qingfeng, Henan	河南清豐
Suiyang, Henan	河南睢陽
Yucheng, Henan	河南虞城
Changshu, Jiangsu	江蘇常熟
Pingxiang, Jiangxi	江西萍鄉
Wuning, Jiangxi	江西武寧
Yujiang, Jiangxi	江西余江
Pingdu, Shandong	山東平度
Fenyang, Shanxi	山西汾陽
Huaxian, Shaanxi	陝西華縣
Jinyan, Sichuan	四川井研
Nanbuxian, Sichuan	四川南部縣
Nanchong, Sichuan	四川南充
Tongnan, Sichuan	四川潼南
Zhongjiang, Sichuan	四川中江
Wuxing, Zhejiang	浙江吳興
Xiangshan, Zhejiang	浙江象山
Zhoushan, Zhejiang	浙江舟山
Dangtu, Anhui	安徽當塗
Jixi, Anhui	安徽績溪
Quanjiao, Anhui	安徽全椒
Tianshui, Gansu	甘肅天水
Yingde, Guangdong	廣東英德
Mengxian, Henan	河南孟縣
Puyang, Henan	河南濮陽
Shanxian, Henan	河南陝縣
Qichun, Hubei	湖北蘄春
Xiangfan, Hubei	湖北襄樊
Rugao, Jiangsu	江蘇如皋
Fuzhou, Jiangxi	江西撫州
Jingdezhen, Jiangxi	江西景德鎮
Yongxin, Jiangxi	江西永新
Yushan, Jiangxi	江西玉山
Laizhou, Shandong	山東萊州
Chongzhou, Sichuan	四川崇州
Pujiang, Sichuan	四川蒲江
Kunshan, Zhejiang	浙江昆山

Shengzhou, Zhejiang	浙江嵊州
Taizhou, Zhejiang	浙江台州
Hechuan, Chongqing	重慶合川
Fuyang, Anhui	安徽阜陽
Guangde, Anhui	安徽廣德
Hefei, Anhui	安徽合肥
Ningguo, Anhui	安徽寧國
Qianshan, Anhui	安徽潛山
Nan'an, Fujian	福建南安
Yongchun, Fujian	福建永春
Yangxin, Hubei	湖北陽新
Xiangyin, Hunan	湖南湘陰
Fengxian, Jiangsu	江蘇豐縣
Liyang, Jiangsu	江蘇溧陽
Lianyungang, Jiangsu	江蘇連雲港
Dean, Jiangxi	江西德安
Duchang, Jiangxi	江西都昌
Fengxin, Jiangxi	江西奉新
Jinxian, Jiangxi	江西進賢
Jiujiang, Jiangxi	江西九江
Yifeng, Jiangxi	江西宜豐
Guang'an, Sichuan	四川廣安
Qingshen, Sichuan	四川青神
Qingtian, Zhejiang	浙江青田
Suichang, Zhejiang	浙江遂昌
Chongqing	重慶
Bozhou, Anhui	安徽亳州
Fengtai, Anhui	安徽鳳台
Wuwei, Anhui	安徽無爲
Yixian, Anhui	安徽黟縣
Guangze, Fujian	福建光澤
Minqing, Fujian	福建閩清
Youxi, Fujian	福建尤溪
Zhengding, Hebei	河北正定
Runan, Henan	河南汝南
Yuzhou, Henan	河南禹州
Changde, Hunan	湖南常德
Hengshan, Hunan	湖南衡山
Qiyang, Hunan	湖南祁陽
Huai'an, Jiangsu	江蘇淮安
Yichun, Jiangxi	江西宜春
Yongxiu, Jiangxi	江西永修

Caoxian, Shandong	山東曹縣
Zibo, Shandong	山東淄博
Yongji, Shanxi	山西永濟
Anyue, Sichuan	四川安岳
Jianyang, Sichuan	四川簡陽
Pixian, Sichuan	四川郫縣
Zizhong, Sichuan	四川資中
Anji, Zhejiang	浙江安吉
Changxing, Zhejiang	浙江長興
Shengxian, Zhejiang	浙江嵊縣
Xuancheng, Anhui	安徽宣城
Fuan, Fujian	福建福安
Jianning, Fujian	福建建寧
Lianxian, Guangdong	廣東連縣
Anyang, Henan	河南安陽
Qinyang, Henan	河南沁陽
Xiangyang, Hubei	湖北襄陽
Yongzhou, Hunan	湖南永州
Jurong, Jiangsu	江蘇句容
Lishui, Jiangsu	江蘇溧水
Wujiang, Jiangsu	江蘇吳江
Xuzhou, Jiangsu	江蘇徐州
Jinxi, Jiangxi	江西金谿
Qianshan, Jiangxi	江西鉛山
Yihuang, Jiangxi	江西宜黃
Xiaxian, Shanxi	山西夏縣
Langzhong, Sichuan	四川閬中
Mianzhu, Sichuan	四川綿竹
Changshan, Zhejiang	浙江常山
Zhaoxian, Hebei	河北趙縣
Ningdu, Jiangxi	江西寧都
Guanghan, Sichuan	四川廣漢
Haiyan, Zhejiang	浙江海鹽
Pujiang, Zhejiang	浙江浦江
Jingxian, Anhui	安徽涇縣
Qujiang, Guangdong	廣東韶關
Zhengzhou, Henan	河南鄭州
Hengyang, Hunan	湖南衡陽
Dantu, Jiangsu	江蘇丹徒
Wuxian, Jiangsu	江蘇吳縣
Shangrao, Jiangxi	江西上饒
Taihe, Jiangxi	江西泰和

Shenxian, Shandong	山東莘縣
Dujiangyan (Guanxian), Sichuan	四川都江堰市（灌縣）
Mianyang, Sichuan	四川綿陽
Leshan, Sichuan	四川樂山
Haining, Zhejiang	浙江海寧
Changting, Fujian	福建長汀
Gutian, Fujian	福建古田
Jinjiang, Fujian	福建晉江
Shaxian, Fujian	福建沙縣
Tongan, Fujian	福建同安
Guangzhou, Guangdong	廣東廣州
Jiangling, Hubei	湖北江陵
Daoxian, Hunan	湖南道縣
Nantong, Jiangsu	江蘇南通
Ganzhou, Jiangxi	江西贛州
Yongfeng, Jiangxi	江西永豐
Qionglai, Sichuan	四川邛崍
Lanxi, Zhejiang	浙江蘭溪
Huian, Fujian	福建惠安
Xuchang, Henan	河南許昌
Qixian, Henan	河南杞縣
Anlu, Hubei	湖北安陸
Danling, Sichuan	四川丹棱
Shuangliu, Sichuan	四川雙流
Kaihua, Zhejiang	浙江開化
Tongxiang, Zhejiang	浙江桐鄉
Yongkang, Zhejiang	浙江永康
Daming, Hebei	河北大名
Suixian, Henan	河南睢縣
Juancheng, Shandong	山東鄄城
Fenghua, Zhejiang	浙江奉化
Lin'an, Zhejiang	浙江臨安
Quxian, Zhejiang	浙江衢縣
Hexian, Anhui	安徽和縣
Shangqiu, Henan	河南商丘
Wujin, Jiangsu	江蘇武進
Dongping, Shandong	山東東平
Taiyuan, Shanxi	山西太原
Fuyang, Zhejiang	浙江富陽
Ninghai, Zhejiang	浙江寧海
Yuhang, Zhejiang	浙江餘杭
Gaoyou, Jiangsu	江蘇高郵

Taizhou, Jiangsu	江蘇泰州
Xingan, Jiangxi	江西新干
Cixi, Zhejiang	浙江慈溪
Yongtai, Fujian	福建永泰
Yixing, Jiangsu	江蘇宜興
Guixi, Jiangxi	江西貴溪
Jinan, Shandong	山東濟南
Qingzhou, Shandong	山東青州
Songjiang, Shanghai	上海松江
Renshou, Sichuan	四川仁壽
Jiande, Zhejiang	浙江建德
Nanping, Fujian	福建南平
Pingjiang, Hunan	湖南平江
Jintan, Jiangsu	江蘇金壇
Anfu, Jiangxi	江西安福
Gaoan, Jiangxi	江西高安
Deqing, Zhejiang	浙江德清
Yuyao, Zhejiang	浙江餘姚
Luoyuan, Fujian	福建羅源
Dexing, Jiangxi	江西德興
Jiangshan, Zhejiang	浙江江山
Tonglu, Zhejiang	浙江桐廬
Xinchang, Zhejiang	浙江新昌
Xuanzhou, Anhui	安徽宣州
Ningde, Fujian	福建寧德
Leping, Jiangxi	江西樂平
Juye, Shandong	山東巨野
Santai, Sichuan	四川三台
Chongren, Jiangxi	江西崇仁
Fengcheng, Jiangxi	江西豐城
Yugan, Jiangxi	江西餘干
Xi'an, Shaanxi	陝西西安
Xiushui, Jiangxi	江西修水
Zhenjiang, Jiangsu	江蘇鎮江
Jishui, Jiangxi	江西吉水
Yiwu, Zhejiang	浙江義烏
Shangyu, Zhejiang	浙江上虞
Xiapu, Fujian	福建霞浦
Dongguan, Guangdong	廣東東莞
Changsha, Hunan	湖南長沙
Xianju, Zhejiang	浙江仙居
Jiangyin, Jiangsu	江蘇江陰

Zhangshu, Jiangxi	江西樟樹
Dongyang, Zhejiang	浙江東陽
Zhangzhou, Fujian	福建漳州
Kunshan, Jiangsu	江蘇昆山
Wuxi, Jiangsu	江蘇無錫
Jiaxing, Zhejiang	浙江嘉興
Quzhou, Zhejiang	浙江衢州
Xinyu, Jiangxi	江西新余
Lishui, Zhejiang	浙江麗水
Wuyuan, Jiangxi	江西婺源
Chun'an, Zhejiang	浙江淳安
Longquan, Zhejiang	浙江龍泉
Ruian, Zhejiang	浙江瑞安
Tiantai, Zhejiang	浙江天台
Fuqing, Fujian	福建福清
Yueqing, Zhejiang	浙江樂清
Jinyun, Zhejiang	浙江縉雲
Boyang, Jiangxi	江西波陽
Xiuning, Anhui	安徽休寧
Pucheng, Fujian	福建浦城
Danyang, Jiangsu	江蘇丹陽
Yangzhou, Jiangsu	江蘇揚州
Linhai, Zhejiang	浙江臨海
Nanchang, Jiangxi	江西南昌
Changle, Fujian	福建長樂
Shexian, Anhui	安徽歙縣
Jianyang, Fujian	福建建陽
Nanfeng, Jiangxi	江西南豐
Changzhou, Jiangsu	江蘇常州
Nanjing, Jiangsu	江蘇南京
Shaowu, Fujian	福建邵武
Huangyan, Zhejiang	浙江黃巖（黃岩）
Linchuan, Jiangxi	江西臨川
Xianyou, Fujian	福建仙遊
Nancheng, Jiangxi	江西南城
Pingyang, Zhejiang	浙江平陽
Wuyishan, Fujian	福建武夷山
Ji'an, Jiangxi	江西吉安
Shaoxing, Zhejiang	浙江紹興
Jinhua, Zhejiang	浙江金華
Meishan, Sichuan	四川眉山
Chengdu, Sichuan	四川成都

Luoyang, Henan	河南洛陽
Huzhou, Zhejiang	浙江湖州
Jian'ou, Fujian	福建建甌
Suzhou, Jiangsu	江蘇蘇州
Quanzhou, Fujian	福建泉州
Wenzhou, Zhejiang	浙江溫州
Putian, Fujian	福建莆田
Hangzhou, Zhejiang	浙江杭州
Fuzhou, Fujian	福建福州
Ningbo, Zhejiang	浙江寧波
Kaifeng, Henan	河南開封
