# Supplementary material for: Visual analysis of geographical distribution of poets in Song China based on Complete Song Poetry
Source: PLoS One. 2024 Sep 6;19(9):e0310115. doi: 10.1371/journal.pone.0310115 (PMC11379205; doi:10.1371/journal.pone.0310115)
Supplement: Attachment — Submitted filename: Response to Reviewers.docx [file pone.0310115.s004.docx]

Dear Editors and Reviewers,

Thank you very much for giving us a valuable opportunity to revise our manuscript, and we really appreciate your significant review assistance for handling our manuscript and sharing the reviewer's comments on our manuscript (ID: PONE-D-24-23728, entitled: “Visual Analysis of Geographical Distribution of Poets in Song China Based on Complete Song Poetry”).

We have carefully studied reviewers’ comments and tried our best to make point-to-point revisions which are marked in red in our marked-up copy. We believe that the revised manuscript would be more reasonable.

The revision reports are listed as following in detail. We hope that the changes can satisfy your requirements. If you have any question, please contact us at any time..

Sincerely yours,

Enhai Lei

School of Chinese Languages and Literatures, Lanzhou University, Lanzhou, P. R. China

**Response to Journal requirements:**

1. When submitting your revision, we need you to address these additional requirements.

Please ensure that your manuscript meets PLOS ONE's style requirements, including those for file naming. The PLOS ONE style templates can be found at 
https://journals.plos.org/plosone/s/file?id=wjVg/PLOSOne_formatting_sample_main_body.pdf and 
https://journals.plos.org/plosone/s/file?id=ba62/PLOSOne_formatting_sample_title_authors_affiliations.pdf.

According to above requirements, the revisions are made as following:

**Revision for title, author and affiliations**:

1) **Page1, line 2 to 4**: The article title “**Visual Analysis of Geographical Distribution of Poets in Song China Based on *Complete Song Poetry***” is changed to “**Visual analysis of geographical distribution of poets in Song China based on *Complete Song Poetry*”.**
2) **Page1, line 6**: “Enhai Lei^1, 2*^, Xudong Hu^1, 2^” is changed to “Enhai Lei^1, 2*¶^, Xudong Hu^1, 2¶^”
3) **Page1, line 8 and 9**: “^1^ School of Chinese Languages and Literatures, Lanzhou University, Lanzhou, 730000, P. R. China; [leieh@lzu.edu.cn](mailto:anything69@163.com) (E.L.); [huxd2013@foxmail.com](mailto:huxd2013@foxmail.com) (X.H.)” is changed to “^1^ School of Chinese Languages and Literatures, Lanzhou University, Lanzhou, P. R. China”
4) **Page 1, line 10**: “Center for Chinese studies, Lanzhou University” is changed to “Center for Chinese Studies, Lanzhou University, Lanzhou, P. R. China”
5) **Page 1, line 11**: “Correspondence: [leieh@lzu.edu.cn](mailto:leieh@lzu.edu.cn)” is changed to “[leieh@lzu.edu.cn](mailto:leieh@lzu.edu.cn) (EL)”
6) **Page 1, line 12**: “¶ These authors contributed equally to this work.” is added.

**Revision for level 1 heading**:

1) **Page 2, line 31**: “**1. Introduction**” is changed to “**Introduction”**
2) **Page 4, line 82 to 83**: “**Research objectives, data collection and processing, and research method**” is added.
3) **Page 7, line 135**: “**Results and discussion**” is added.
4) **Page 24, line 493**: “**4. Conclusion**” is changed to “**Conclusion**”.

**Revision for level 2 heading**:

1) **Page 4, line 84**: “**Research objectives**” is added.
2) **Page 5, line 98**: “**Data sources and processing**” is added.
3) **Page 6, line 113**: “**Research method**” is added.
4) **Page 7, line 136 to 137**: “**2. The recordings of poets' native place in Complete Song Poetry and their characteristics of geographical distribution**” is changed to “**The recordings of poets' native place in Complete Song Poetry and their characteristics of geographical distribution**”.
5) **Page 13, line 280 to 281**: “**3. The formation reasons behind the geographical distribution characteristics of poets in the Complete Song Poetry**” is changed to “**The formation reasons behind the geographical distribution characteristics of poets in the Complete Song Poetry**”.

**Revision for level 3 heading**:

1) **Page 15, line 322**: “**3.1 The distribution of Guangdong poets and its formation causes**” is changed to “**The distribution of Guangdong poets and its formation causes**”.
2) **Page 21, line 426 to 427**: “**3.2 The distribution of poets in Ningbo, Luoyang and Meishan, and their formation causes**” is changed to “**The distribution of poets in Ningbo, Luoyang and Meishan, and their formation causes**”.

**Revision for Acknowledgments**:

**Page 24, line 506 to 509**: “Acknowledgments: We sincerely acknowledge for Prof. Zhibing Zhang′s advice for our manuscript. We also thank Xuewei Zhao and Xueli Feng for their assistance during the preparation of our manuscript.” is added.

**Revision for Author Contributions**:

“E.L. proposed the research ideas and methods of the manuscript; X.H. was responsible for data collection, writing and creating the figures and forms; All authors have read and agreed to the published version of the manuscript.” is deleted. The author contributions have been selected in submission system as following:

“Conceptualization: Enhai Lei.

Data Curation: Xudong Hu.

Formal Analysis: Xudong Hu.

Funding Acquisition: Enhai Lei.

Investigation	: Xudong Hu.

Methodology: Enhai Lei.

Project Administration: Enhai Lei.

Resources: Xudong Hu.

Software: Xudong Hu

Supervision: Enhai Lei

Validation: Enhai Lei.

Visualization: Xudong Hu.

Writing – Original Draft Preparation: Xudong Hu.

Writing – Review & Editing: Enhai Lei, Xudong Hu.”.

2. Thank you for stating the following financial disclosure: 
 [This research was funded by the Gansu Education Department (2022CYZC-09).].  
Please state what role the funders took in the study.  If the funders had no role, please state: ""The funders had no role in study design, data collection and analysis, decision to publish, or preparation of the manuscript."" 
If this statement is not correct you must amend it as needed. 
Please include this amended Role of Funder statement in your cover letter; we will change the online submission form on your behalf.

**Revision for Funding**:

The sentence “**Funding:** This research was funded by the Gansu Education Department (2022CYZC-09).” is deleted in the manuscript. The funding statement “This research was funded by the Gansu Education Department (Grant No. 2022CYZC-09). Enhai Lei received the funded project. The funders had no role in study design, data collection and analysis, decision to publish, or preparation of the manuscript. Website of the funder: <http://jyt.gansu.gov.cn/>” is put in our cover letter. Please replace it for us in the submission system.

3. Please include captions for your Supporting Information files at the end of your manuscript, and update any in-text citations to match accordingly. Please see our Supporting Information guidelines for more information: http://journals.plos.org/plosone/s/supporting-information.

**Revision for Supporting information**:

**Page 27, line 581 to 585**, the captions for our Supporting Information files are added at the end of our manuscript as following:

“Supporting information

S1 Dataset. Database of Poets' native places in the Complete Song Poetry.

S2 Dataset. Database of Gangdong Poets' native places in the Complete Song Poetry.

S1 File. Supporting Information for references, citations and figures.”.

The in-text citations have been revised to match accordingly.

**Response to Reviewer #1:**

1. **Question:**

The study utilizes GIS software and a comprehensive dataset, which is a commendable approach. However, the methodology section lacks sufficient detail on data processing, GIS visualization steps, and statistical analysis. Inclusion of this information would enhance the technical rigor and reliability of the study.
**Suggestions**: Add detailed descriptions of the GIS data processing methods, statistical tests used, and how data accuracy was ensured.

**Reply:**

**Page 4 to 7, line 82 to 133:** A new section “**Research objectives, data sources, data processing, and research method**” is added after the **Introduction** section. In this new section, the research objectives, the GIS data collection and processing, and research method are described. This section is added as following:

**Research objectives, data collection and processing, and research method**

**Research objectives**

Building on the above findings, the previous studies on the geographical distribution of poets during the Song Dynasty involved about a thousand individuals. In fact, the *Complete Song Poetry* documents over 9000 poets from the Song Dynasty in its total of 72 volumes, almost encompassing all of the poets in Song Dynasty and offering a comprehensive overview of Song Dynasty poets. Therefore, studying on the geographical distribution of all poets based on the *Complete Song Poetry* is essential for a comprehensive understanding of poets' distribution in Song Dynasty, and then offering more accurate insight into the regional characteristics of literature in Song Dynasty. In this work, we will perform a visualized analysis of geographical distribution of all Song poets with clear native places based on the *Complete Song Poetry*, and discuss its formation reasons to provide a comprehensive understanding of geographical distribution of Song poets and its relation with the development of Song Dynasty society.

**Data sources and processing**

The *Complete Song Poetry* contains over 250,000 Song poems. Due to the massive number of poems, the Department of Chinese Language and Literature of Peking University developed the *Complete Song Poetry Analysis System* [7] in 2005, which support full text search, poet biography search and advanced search. This system will be instrumental in collecting and processing the extensive and intricate data of poets with clear native place records, enabling quantitative analysis of their geographical distribution through statistical method. This system ensures the accuracy of the data we obtain, and a total of valid 9200 poet's data were collected from the system, with 6,056 poets having clear known native place and 3,144 poets having no clear native place, which provides a basic data guarantee for our subsequent research work.

To achieve data visualization, we use Tianditu (Map World) [8] to obtain accurate latitude and longitude coordinates for each location of poet's native place. Furthermore, the data of 6,056 poets with clear known native place was imported into GIS software to present the geographical distribution of poets.

**Research method**

**Spatial query and measurement.** Spatial query, one of the basic functions of GIS, allows users to filter and query spatial objects based on geographic location, attributes, and other conditions. Through spatial queries, data about the corresponding objects can be obtained for further detailed analysis. Spatial measurement is to measure the spatial attributes of geographic objects, which allow us to better understand the spatial distribution characteristics and relationships of geographic elements. This study will utilize spatial query and measurement to spatialize the collected data of poets' native places to present their distribution on the map.

**Jenks natural breaks optimization.** This method aims to determine the best arrangement of data into different classes, which is particularly effective for classifying data with uneven distribution. In this study, Jenks natural breaks optimization has been used to achieve effective stratification for the massive data of poets' native places.

**Graduated Symbols Renderer.** The graduated symbol renderer is a sophisticated tool for representing quantitative data, excelling in categorizing quantitative data into ordered classes, with each class distinguished by a consistent set of symbols. The system applies a series of graduated symbols to each class, incrementally enhancing the visual representation. This study applies the graduated symbol renderer to process visually the data of poets' native place, enabling it to be presented more accurately on the map and allowing for a more intuitive understanding of the poets' geographic distribution.

2. **Question:**

Introduction Section Review
Current Introduction:

Paragraph 1:

"China is a country of poetry, and after thriving of poetry in Tang Dynasty, the Song China (960-1279) saw another summit in the development of Chinese poetry. Thousands of poets were appeared during this time and scattered across China."
**Comment:** This opening sets the context well but could be smoother. It should also mention the importance of understanding the geographical distribution of these poets.

**Reply:**

**Page 2, line 34 to 35:** According to this comment, the sentence “The geographical distribution of poets is closely related to regional social development.” is added.

3. **Question:**

Paragraph 2:
"Studying the geographical distribution of poets in Song Dynasty can get insights into the cultural, economic, and political development of Song Dynasty society. From statistical and geographical distribution perspectives, the research works on the distribution of poets in the Song Dynasty have been performed previously in the academics, resulting many research achievements."
**Comment:** This paragraph introduces the significance of the study and acknowledges prior research. However, it could transition more smoothly by linking the historical context to the need for this study.

**Reply:**

**Page 2, line 36 to 38:** After description of importance for understanding the geographical distribution of these poets. The original sentence “Studying the geographical distribution of poets in Song Dynasty can get insights into the cultural, economic, and political development of Song Dynasty society.” is changed to “Consequently, studying on the geographical distribution of poets in Song Dynasty can get insights into the cultural, economic, and political development of Song Dynasty society”.

**Page 2, line 40 to 43:** To make transition more smoothly, the description “Up to now, there are two research methods used to study the geographical distribution of poets in Song Dynasty. One is to investigate the geographical distribution of poets from a statistical perspective, while another tries to present it in visual methods. For instance, as far back as in the 1990s,” is added.

4. **Question:**

Paragraph 3:
"As far back as the 1990s, Zeng Daxing's book 'Geographical Distribution of Chinese Literary Masters of Previous Dynasties' [1] presented a statistical analysis of the geographical distribution of ancient Chinese literary masters. In chapter 6 of this book, the geographical distribution patterns of literary scholars in Song, Liao, and Jin Dynasties along with their formation reasons were discussed based on the number of writers’ native places recorded in Tan Zhengbi’s 'Dictionary of Chinese Literati' [2], and 1168 poets were involved in total."
**Comment:** This paragraph delves into specific prior works. It transitions from general significance to specific examples, which is logical but could benefit from a clearer linkage.

**Reply:**

**Page 2, line 40 to 43:** To make a clearer linkage from general significance to specific examples, a summarized description of previous research methods “Up to now, there are two research methods used to study the geographical distribution of poets in Song Dynasty. One is to investigate the geographical distribution of poets from a statistical perspective, while another tries to present it in visual methods.” and “For instance” is added before “As far back as the 1990s, Zeng Daxing's book…”.

**Page 3, line 50 to 52:** A comment to Zeng's work “Zeng's work simply analyzed the geographical distribution of Song poets statistically in the form of tables, and examined the correlation between the distribution of poets and the local culture.” is added as research background to evaluate Zeng's contribution and deficiency in this research field.

5. **Question:**

Paragraph 4:
"In 2003, Wang Zhaopeng and Liu Xue published a paper entitled “Statistical Analysis of Poets in Song Ci” [3], in which a statistical analysis of 880 poets in Song Dynasty was performed, analyzing their geographical and temporal distributions. They concluded that the development of Song Ci, a special type of poem in Song Dynasty, was intimately linked to the prosperity of Song poetry."
**Comment:** This continuation of prior works is consistent with the previous paragraph. Ensure smooth transition by summarizing how these works build a foundation for the current study.
**Reply:**

**Page 3, line 52 to 55:** In order to give a smoother transition, the sentence “In 2003, Wang Zhaopeng and Liu Xue published a paper entitled “Statistical Analysis of Poets in Song Ci” [3], in which a statistical analysis of 880 poets in Song Dynasty was performed, analyzing their geographical and temporal distributions.” is changed to “Afterwards, Wang Zhaopeng and Liu Xue published a paper in 2003, entitled “Statistical Analysis of Poets in Song Ci” [3], in which a statistical analysis of 880 poets in Song Dynasty was performed to present their geographical and temporal distributions.”.

**Page 3, line 57 to 59:** A comment to Wang and Liu's work “Comparing to Zeng′s research work, Wang and Liu's work, combining the economy, politics, and population factors of the Song Dynasty, provides a more detailed geographical distribution of poets in the Song Dynasty.” is added as research background to evaluate Wang and Liu's contribution in this research field.

6. **Question:**

Paragraph 5:
"In Lou Xinxing’s article “Geographical Distribution of Authors in Complete Song Iambic Verse Collection and the Cause” [4], a statistical and visual analysis about the distribution of poets in Complete Song Ci at the provincial and county levels was performed, with a particular focus on poets from Zhejiang province."
**Comment:** Maintains consistency in reviewing past research. Ensure to link it back to the current study's objectives for better coherence.
**Reply:**

**Page 3 to 4, line 59 to 70:** Lou Xinxing first used visual analysis method to the study of the distribution of poets in Complete Song Ci. In order to maintain consistency in reviewing past research and ensure to link it back to the current study's objectives for better coherence. The modified paragraph “In 2020, Lou Xinxing visually presented the distribution of poets in *Complete Song Ci* at the provincial and county levels in her article “Geographical Distribution of Authors in *Complete Song Iambic Verse Collection* and the Cause” [6], with particularly focusing on poets from Zhejiang province.” is moved behind the modified paragraph “Additionally, Wang Xiang's “Analysis of the Geographical Distribution of Poets in Northern Song Dynasty and Their Significance in Literary History” [4] in 2006 recorded the geographical distribution of 975 poets in the Northern Song Dynasty based on *Complete Song Poetry* [5], which was compiled by the Center for Ancient Chinese Classics and Archives of Peking University and published by Peking University Press in 1998. He divided the Northern Song Dynasty into four stages from a literary history perspective to discuss the geographical distribution of poets, providing insights into the literary prosperity and cultural center of the Song Dynasty.”

**Page 4, line 70 to 75:** A comment to Lou Xinxing's work “She is one of the few scholars in recent years to utilize visualization methods to study the geographical distribution of poets in Song Dynasty. She has drawn several maps, and intuitively and clearly show the geographical distribution of poets in the Song Dynasty. However, only 820 Song poets were involved in her research work, which did not cover the majority of poets in Song Dynasty.” is added as research background to evaluate Lou Xinxing's contribution in this research field.

7. **Question:**

Paragraph 6:
"Additionally, Wang Xiang's 'Analysis of the Geographical Distribution of Poets in Northern Song Dynasty and Their Significance in Literary History' [5] recorded the geographical distribution of 975 poets in the Northern Song Dynasty based on Complete Song Poetry, which was compiled by the Center for Ancient Chinese Classics and Archives of Peking University and published by Peking University Press in 1998. He divided the Northern Song Dynasty into four stages from a literary history perspective to discuss the geographical distribution of poets, providing insights into the literary prosperity and cultural center of the Song Dynasty."
**Comment:** Another specific example of prior work. Transition is logical but could be enhanced by summarizing the cumulative insights these studies provide for the current research.
**Reply:**

Wang Xiang still used the statistical method in his research work as before. However, Lou Xinxing first used visualization methods in her study. To correspond with our research, the description about Wang Xiang's work was moved before Lou Xinxing’s work. It is presented as in the **Reply** of **Question 6**.

**Page 4, line 76 to 80:** To summarizing the cumulative insights provided by the previous studies for the current research, the Paragraph “Overall, scholars have achieved significant research results on the geographical distribution of poets in the Song Dynasty in terms of statistics. However, studies employing visualized presentations of the poet's distribution are relatively scarce, and the poets involved in such research works are only around a thousand people, which did not fully cover the poets in Song Dynasty.” is added at the end of the **Introduction**.

8. **Question:**

Paragraph 7:
"In a word, the previous studies on the geographical distribution of poets during the Song Dynasty involved about a thousand individuals. In fact, the Complete Song Poetry documents over 9000 poets from the Song Dynasty in its total of 72 volumes, offering a comprehensive overview of Song Dynasty poets. Therefore, studying on the geographical distribution of all poets in the Complete Song Poetry is essential for a comprehensive understanding of poets' distribution in Song Dynasty, and then offering more accurate insight into the regional characteristics of literature in Song Dynasty."
**Comment:** This paragraph effectively summarizes the gaps in the existing literature and sets the stage for the current study. The transition here is smooth and logical.
**Reply:**

**Page 4 to 5, line 85 to 97:** This paragraph describes actually the research objectives and reason of our research work. Thus, it was moved to the new section ‘**Research objectives**’ in our revised manuscript as the following modified paragraph:

“Building on the above findings, the previous studies on the geographical distribution of poets during the Song Dynasty involved about a thousand individuals. In fact, the *Complete Song Poetry* documents over 9000 poets from the Song Dynasty in its total of 72 volumes, almost encompassing all of the poets in Song Dynasty and offering a comprehensive overview of Song Dynasty poets. Therefore, studying on the geographical distribution of all poets based on the *Complete Song Poetry* is essential for a comprehensive understanding of poets' distribution in Song Dynasty, and then offering more accurate insight into the regional characteristics of literature in Song Dynasty. In this work, we will perform a visualized analysis of geographical distribution of all Song poets with clear native places based on the *Complete Song Poetry*, and discuss its formation reasons to provide a comprehensive understanding of geographical distribution of Song poets and its relation with the development of Song Dynasty society.”

**Page 5, line 93 to 97:** To give clearer summarization of our research objective and enhance the smoothness of transition, the sentence “In this work, we will perform a visualized analysis of geographical distribution of all Song poets with clear native places based on the *Complete Song Poetry*, and discuss its formation reasons to provide a comprehensive understanding of geographical distribution of all Song poets and its relation with the development of Song Dynasty society.” is added at the end of this paragraph.

9. **Question:**

Paragraph 8:
"Given the massive number of poets in the Complete Song Poetry, the Complete Song Poetry Analysis System, [6] which is developed by the Department of Chinese Language and Literature of Peking University in 2005, will be instrumental in collecting and processing the extensive and intricate data of poets with clear native place records, enabling quantitative analysis of their geographical distribution through statistical method. In particular, GIS software will be used herein to visually display the geographical distribution and density of the poets in Complete Song Poetry to explore the characteristics of the geographical distribution of poets and the formation reasons behind it, and thus making progress for the research works on the investigation of the geographical distribution of poets from the Song Dynasty."
**Comment:** This final paragraph clearly outlines the methodology and tools used in the current study, effectively transitioning from the literature review to the research methodology.
**Reply:**

This paragraph outlines actually the data collecting, data processing, methodology and tools used in our study. Thus, two new section ‘**Data sources and processing**’ and ‘**Research method**’ is added in our revised manuscript.

The following modification of this paragraph is added as ‘**Data sources and processing’** section in **Page 5 to 6, line 98 to 112**.

“**Data sources and processing**

The *Complete Song Poetry* contains over 250,000 Song poems. Due to the massive number of poems, the Department of Chinese Language and Literature of Peking University developed the *Complete Song Poetry Analysis System* [7] in 2005, which support full text search, poet biography search and advanced search. This system will be instrumental in collecting and processing the extensive and intricate data of poets with clear native place records, enabling quantitative analysis of their geographical distribution through statistical method. This system ensures the accuracy of the data we obtain, and a total of valid 9200 poet's data were collected from the system, with 6,056 poets having clear known native place and 3,144 poets having no clear native place, which provides a basic data guarantee for our subsequent research work.

To achieve data visualization, we use Tianditu (Map World) [8] to obtain accurate latitude and longitude coordinates for each location of poet's native place. Furthermore, the data of 6,056 poets with clear known native place was imported into GIS software to present the geographical distribution of poets.”

Another new section of ‘**Research method’** is added in **Page 6 to 7, line 113 to 133**, which is presented as following:

**Research method**

**Spatial query and measurement.** Spatial query, one of the basic functions of GIS, allows users to filter and query spatial objects based on geographic location, attributes, and other conditions. Through spatial queries, data about the corresponding objects can be obtained for further detailed analysis. Spatial measurement is to measure the spatial attributes of geographic objects, which allow us to better understand the spatial distribution characteristics and relationships of geographic elements. This study will utilize spatial query and measurement to spatialize the collected data of poets' native places to present their distribution on the maps.

**Jenks natural breaks optimization.** This method aims to determine the best arrangement of data into different classes, which is particularly effective for classifying data with uneven distribution. In this study, Jenks natural breaks optimization has been used to achieve effective stratification for the massive data of poets' native places.

**Graduated Symbols Renderer.** The graduated symbol renderer is a sophisticated tool for representing quantitative data, excelling in categorizing quantitative data into ordered classes, with each class distinguished by a consistent set of symbols. The system applies a series of graduated symbols to each class, incrementally enhancing the visual representation. This study applies the graduated symbol renderer to process visually the data of poets' native place, enabling it to be presented more accurately on the map and allowing for a more intuitive understanding of the poets' geographic distribution.

10. **Question:**

Suggested Improvements for Transitions:
Smooth Transitions: To enhance the smoothness of transitions, consider adding brief summary sentences at the end of each paragraph that link to the next one. This can help guide the reader through the narrative logically.
Consistent Linking: Ensure each paragraph explicitly connects back to the central research question of the current study. This can be done by emphasizing how each piece of prior research contributes to understanding the broader topic.
Cohesive Flow: Use transition phrases like "Building on these findings," "In continuation," "Expanding upon," and "Furthermore," to improve the flow between paragraphs.
**Reply:**

Based those suggestions, the following changes have been made:

1) **Page 2, Line 40 to 43**: For a smooth transition, the sentence “Up to now, there are two research methods used to study the geographical distribution of poets in Song Dynasty. One is to investigate the geographical distribution of poets from a statistical perspective, while another tries to present it in visual methods.” is added. “as far back as…” is changed to “For instance, as far back as…”.
2) **Page 18, Line 365-368**: For a smooth transition, the sentences “These are also clearly recorded in “Difangzhi”, which is also translated as “local gazetteers” [13]. In the following, we will present some formation reasons about geographical distribution of Guangdong poets in Song China illustrated by “local gazetteers” and other related historical records.” is added.
3) For a smooth transition, transition phrases including “Over time” (page 1, line 21), “Consequently” (page 2, line 36), “Up to now” (page 2, line 40), “For instance” (page 2, line 43), “Afterwards,” (page 3, line 52) “Overall” (page 4, line 76), “Building on the above findings” (page 4, line 85), and so on, are added.

The other Changes can also refer to **Reply** of **Question 13**.

11. **Question:**

Statistical Analysis
Statistical Analysis: No
The manuscript should include comprehensive descriptions of the statistical methods utilized to validate the findings. Thorough statistical analysis is imperative for establishing the credibility and robustness of the results.
**Suggestions**: In your analysis, please provide a detailed description of the statistical methods utilized, including the specific tests applied, significance criteria, and data handling procedures. Additionally, discuss the validation of results and any replication or controls utilized in the study.
**Reply:**

A new section ‘**Research objectives, data sources, data processing, and research method**’ is added **from Page 4 to 7, line 82 to 133**. In this section, data collection, data processing and research methods is described in detail. Especially, statistical methods, including ‘**Spatial query and measurement’**, **‘Jenks natural breaks optimization’** and **‘Graduated Symbols Renderer’** used in our work were added.

The ‘**Spatial query and measurement’** is used to spatialize the collected data of poets' native places to present their distribution on the maps.

The **‘Jenks natural breaks optimization’** is used to achieve effective stratification for the massive data of poets' native places.

The **‘Graduated Symbols Renderer’** is used to the visualization process of poets' native place data, enabling it to be presented more accurately on the map and allowing for a more intuitive understanding of the poets' geographic distribution.

12. **Question:**

Data Availability
Data Availability: Yes
The authors have ensured that all pertinent data supporting their findings are included in the manuscript and its accompanying information files. This practice aligns with PLOS ONE's data policy, which emphasizes transparency and the ability to reproduce the results.
No further action needed.
**Reply:**

We have carefully verified all the data again and supplemented some missing place names in Table 1, including Meizhou, Yangjiang, Suixi and Shenzhen. The Table is remade based on the revised dataset, which can be found in S2 Dataset of supporting information. A legend for Table 1 is added in **Page 17, Line 338 to 339**. The Fig 4 is redrew based on this information.

In addition, all raw data was placed in the supporting materials S1 dataset and S2 dataset, including information about place names, geographic coordinates, amount of poets.

13. **Question:**

Presentation and Language
Presentation and Language: Yes
The manuscript demonstrates strong structural organization and employs standard English conventions. Nonetheless, it contains minor grammatical and typographical errors that warrant attention to enhance overall readability.
Suggestions: Conduct a thorough proofread to correct grammatical errors and awkward phrasings. Examples include:
Abstract: "With the time going on" → "Over time"
Introduction: "after thriving of poetry in Tang Dynasty" → "after the thriving of poetry in the Tang Dynasty"
Results: "data from Fig 1 reveals" → "data in Fig 1 reveals"
**Reply:**

1) **Page 1, Line 21**: The phrase “With the time going on” is changed to “Over time”.
2) **Page 2, Line 32 to 33**: “after thriving of poetry in Tang Dynasty” is changed to “after the thriving of poetry in Tang Dynasty”.
3) **Page 2, Line 32 and 33**: “(618-907)” is added after “Tang Dynasty”
4) **Page 2, Line 34**: “Thousands of poets were appeared…” is changed to “Thousands of poets appeared…”.
5) **Page 2, Line 36**: “Studying on…” is changed to “Consequently, studying on…”.
6) **Page 2, Line 40**: “resulting” is changed to “resulting in”.
7) **Page 2, Line 43**: “As far back as 1990s,…” is changed to “For instance, as far back as in the 1990s,…”.
8) **Page 3, Line 52 to 55**: “In 2003, Wang Zhaopeng and Liu Xue published a paper entitled “Statistical Analysis of Poets in Song Ci” [3], in which a statistical analysis of 880 poets in Song Dynasty was performed, analyzing their geographical and temporal distributions” is changed to “Afterwards, Wang Zhaopeng and Liu Xue published a paper in 2003, entitled “Statistical Analysis of Poets in Song Ci” [3], in which a statistical analysis of 880 poets in Song Dynasty was performed to present their geographical and temporal distributions.”.
9) **Page 3, Line 61**: “in 2006” is added after “Wang Xiang's “Analysis of the Geographical Distribution of Poets in Northern Song Dynasty and Their Significance in Literary History” [4]”.
10) **Page 4, Line 70**: “with a particular focus on” is changed to “with particularly focusing on”.
11) **Page 4, Line 67 to 70**: “In Lou Xinxing’s article “Geographical Distribution of Authors in *Complete Song Iambic Verse Collection* and the Cause” [4], a statistical and visual analysis about the distribution of poets in *Complete Song Ci* at the provincial and county levels was performed, with a particular focus on poets from Zhejiang province.” is changed to “In 2020, Lou Xinxing visually presented the distribution of poets in *Complete Song Ci* at the provincial and county levels in her article “Geographical Distribution of Authors in *Complete Song Iambic Verse Collection* and the Cause” [6], with particularly focusing on poets from Zhejiang province.”.
12) **Page 4, Line 85**: “In a word,..” is changed to “Building on the above findings,..”.
13) **Page 4 and 5, Line 88 to 89**: “offering a comprehensive overview of Song Dynasty poets…” is changed to “almost encompassing all of the poets in Song Dynasty and offering a comprehensive overview of Song Dynasty poets...”.
14) **Page 5, Line 90**: “in the *Complete Song Poetry*” is changed to “based on the *Complete Song Poetry*”.
15) **Page 5, Line 99-103**: “Given the massive number of poets in the *Complete Song Poetry*, the *Complete Song Poetry Analysis System*, [6] which is developed by the Department of Chinese Language and Literature of Peking University in 2005, will be…” is changed to “The *Complete Song Poetry* contains over 250,000 Song poems. Due to the massive number of poems, the Department of Chinese Language and Literature of Peking University developed the *Complete Song Poetry Analysis System* [7] in 2005, which support full text search, poet biography search and advanced search. This system will be…”.
16) **Page 7, Line 135**: “**Results and Discussions**” is added.
17) **Page 7, Line 152**: “(Jukju, or Pyeongju, Korea)” is changed to “(Jukju or Pyeongju, Korea)”.
18) **Page 8, Line 159 to 162**: “Yingzhi participated in and passed the examination of Xianliang Fanfzheng Zhiyan Jikjian Ke [7], and was awarded Mishusheng Zhuzuolang [8].” is changed to “Yingzhi participated in and passed the examination of Xianliang Fanfzheng Zhiyan Jikjian Ke (an examination on virtuous and upright officials giving candid advice) and was awarded Mishusheng Zhuzuolang (an assistant officer in the secretariat)”.
19) **Page 8, Line 169**: “…have no native place” is changed to “…have no clear native place”.
20) **Page 8, Line 171**: “exceluding” is changed to “excluding”.
21) **Page 10, Line 208**: “data from Fig 1 reveals” is changed to “data in Fig 1 reveals”
22) **Page 10, Line 219**: “out-standing” is changed to “outstanding”
23) **Page 15, Line 309**: “(Fig 1)” is added.
24) **Page 16, Line 332**: “distribution Table of Guangdong poets.” is changed to “distribution of Guangdong poets.”
25) “QGIS software” in **Page 11, Line 230** and **Page 16, Line 333** are changed to “GIS software”.
26) **Page 17, Line 349**: “for” is changed to “of”
27) **Page 17, Line 354 to 356**: “the Song government paid great importance to foreign maritime trade.” is changed to “the Song government paid great importance to foreign maritime trade, and made many liberal policies to promote foreign trade [12].”
28) **Page 18, Line 360**: “GuangZhou Sibosi” is changed to “Guangzhou Shibosi”
29) **Page 19, Line 382**: “states” is changed to “stated”.
30) **Page 21, Line 430**: “is” is changed to “was”.”
31) **Page 21, Line 446**: “Mingzhoushibosi” is changed to “Mingzhou Shibosi
32) **Page 22, Line 463**: “,” is changed to “:”.
33) **Page 23, Line 472**: “farther of female poet” is changed to “father of the female poet”.
34) **Page 23, Line 483**: “within the basin” is changed to “here”.
35) **Page 23, Line 489**: “Meishan phenomenon” is changed to “Meishan immigration phenomenon”.
14. **Question:**

Implications and Discussion
Implications and Discussion: Needs Improvement
The results are well organized, but the discussion section needs to delve deeper into the wider implications of the findings. This should encompass their influence on our comprehension of Song Dynasty society and the variables affecting the geographic spread of poets.
**Suggestions**: Discuss the socio-political and economic contexts that may have influenced the distribution patterns. Address any limitations of the study and potential biases in the data.
**Reply:**

According to suggestion, the following paragraph is added:

**Page 13 to 14, Line 285 to 294**: “Taking Kaifeng and Hangzhou as examples, they are ranked first and fourth in Fig. 2, respectively. Since it was established as the Northern Song capital in 960 AD, Kaifeng became a combination of imperial space and commercial space, gathering a large amount of wealth [10]. Many high-ranking imperial officials who were also outstanding poets lived here. After Kaifeng was captured by the Jin army in 1127 AD, a large number of royal members and scholar-officials fled south and established a new capital in Hangzhou. Patriotic poets also gathered in this city with the magnificent scenery of West Lake, and accused the fall of the north and the inaction of the authorities with poetry [11]. On the contrary, taking Hainan as an example, it was a remote place used to exile prisoners during the Song Dynasty. Therefore, few poets were distributed here.”

15. **Question:**

Clarity of Figures
Clarity of Figures: Needs Improvement
The visual representations, such as figures and maps, aid in articulating the discoveries, although some require enhanced clarity and readability. It is essential to augment the resolution and labeling in order to proficiently communicate the data.
Suggestions: Please enhance the clarity and readability of all figures by increasing the resolution, improving labeling, and providing more detailed captions to guide the reader.
**Reply:**

We have remade all the Figures in our work to meet PlosOne's requirements for clarity. The presentation format of Fig 1B has been modified to present the data better.

**The original Fig 1B**

**The current Fig 1**

The original Fig 3-6 are merged into the current Fig 3 to present the geographical distribution of the poets more intuitively.

**The original Fig 3-6:**

original Fig 3

original Fig 4

original Fig 5

original Fig 6

**The current Fig 3:**

Accordingly, the original Figs 7 and 8 are changed to the current Fig 4 and 5.

**The original Figs 7**

**The current Fig 4**

**The original Figs 8**

**The current Fig 5**

16. **Question:**

Additional Comments:
Figures and Visualizations:
Please ensure that the figures and maps are accompanied by comprehensive and descriptive captions, along with in-text explanations to maximize their utility. Additionally, make sure that each figure is unambiguously referenced and discussed within the main body of the text.
**Reply:**

Descriptive legends for Fig 1-5 are changed as following:

1) **Page 9, Line 178 to 180**: The sentence “This figure shows the ranking of the poets' geographical distribution on provincial level as well as the percentage of poets in each province.” is added to legend of Fig 1.
2) **Page 9, Line 183 to 184**: The sentence “Due to the massive data, this figure only lists the top 50 specific locations. The entire data see S1 Dataset.” is added to legend of Fig 2.
3) **Page 11 to 12, Line 242 to 245**: The legend of Fig 3 is changed to “(A) Distribution in entire country. (B) North Central China Core. (C) Southwest Inland Core. (D) Southeast Coastal Core. Coordinates of the poet's native place see S1 Dataset. The base map was obtained from Natural Earth [9]. Map credit: Xudong Hu.”
4) **Page 17, Line 342**: The phrase “The detail data see S2 Database.” is added to legend of Fig 4.
5) **Page 17, Line 345 to 346**: The sentence “Coordinates of the poet's native place see S2 Dataset. The base map was obtained from Natural Earth [9]. Map credit: Xudong Hu.” is added to legend of Fig 5.

The in-text explanations and references of Fig 1-5 have been checked carefully.

17. **Question:**

References:
The references are cited correctly and written in a standard format. However, make sure all sources are accurately cited and consistently formatted throughout the manuscript.
However, ensure all references consistently include:
Author(s)
Year of publication
Title of the work (italicized for books and journal names, with proper capitalization)
Publisher or journal name
Volume and issue number (for journal articles)
Page numbers (if applicable)
Make sure to check for any inconsistencies in the format throughout the manuscript to maintain uniformity.
**Reply:**

According to the reviewer′s comment and the requirements of PLOS ONE, the references have been carefully examined and written in standard format. The new reference list is as following:

1. Zeng DX. *Zhongguo lidai wenxuejia zhi dilifenbu* (Geographical Distribution of Chinese Literary Masters of Previous Dynasties). Wuhan: Hubei jiaoyu chubanshe (Hubei Education Press); 1995.

2. Tan ZB. *Zhongguo wen xue jia da ci dian* (Dictionary of Chinese Literati). Shanghai: Shanghai shudian (Shanghai Bookstore Publishing House); 1981.

3. Wang ZP, Liu X. *Songci zuozhe tongji fenxi* (Statistical Analysis of Authors in Song Ci). Literature & Art Studies*.* 2003; 06: 54-59.

4. Wang X. *Beisong Shiren de dilifenbu jiqi wenxuyeshi yiyifenxi* (The Gographical Distribution of Poets in the Northern Song Dynasty and Its Literary Significance). Literary Heritage. 2006; 06: 52-62.

5. Center for Ancient Chinese Classics and Archives of Peking University, ed. *Quansongshi*(Complete Song Poetry).Peking University Press. 1998.

6. Lou XX. *Quansongci zuozhe de dilifenbu jiqi chengyin* (Geographical Distribution of Authors in *Complete Song Iambic Verse Collection* and the Cause). Journal of Taizhou University. 2020; 42(02): 57-62. <https://link.oversea.cnki.net/doi/10.13853/j.cnki.issn.1672-3708.2020.02.012>

7. Department of Chinese Language and Literature of Peiking University; 2005[cited 4 Feb 2024] Complete Song Poetry Analysis System. <http://www.chinabooktrading.com/song>

8. National Platform for Common GeoSpatial Information Services. Tianditu (Map Word). National Geomatics Center of China; 2024. <https://map.tianditu.gov.cn>

9. Made with Natural Earth. Free vector and raster map data @ naturalearthdata.com. <https://www.naturalearthdata.com/about/terms-of-use/>

10. Christian de P. *Purchase on Power: Imperial Space and Commercial Space in Song-Dynasty Kaifeng, 960-1127*. Journal of the Economic and Social History of the Orient. 2009; 53(1-2): 149-184. <https://doi.org/10.1163/002249910X12573963244322>

11. Xinda L. *How Does an Objective Correlative Objectify? West Lake as the Site for Patriotic Sentiment in Southern Song Lyrics.* In Joseph SC L, Shuen-fu L, Christian de P, et al. *Senses of the City: Perceptions of Hangzhou and Southern Song China, 1127–1279*. The Chinese University of Hong Kong Press, 2017. pp. 205-233. <https://doi.org/10.2307/j.ctv2n7r8r>

12. Chan KS. *Foreign trade, commercial policies and the political economy of the Song and Ming dynasties of China*. Australian Economic History Review, 2008; 48(1): 68-90. <https://doi.org/10.1111/j.1467-8446.2007.00224.x>

13.James MH. *Song Dynasty Local Gazetteers and Their Place in The History of Difangzhi Writing*. Harvard Journal of Asiatic Studies, 1996; 56(2): 405–442. <https://doi.org/10.2307/2719404>

14. Li MY. *Chongxiu Nanhai Xianzhi Xu* (Preface to the Revised Gazetteer of Nanhai County). In Dai ZC, Shi C, eds. *Guangzhoufuzhi* (Gazetteer of Guangzhou Prefecture), Guangzhou: Yuexiu Shuyuan. 1879; vol.15. <http://read.nlc.cn/OutOpenBook/OpenObjectBook?aid=403&bid=81365.0>

15. Wang ZX. *Qianxueji* (Relocation Record of School) In Dai ZC, Shi C, eds. *Guangzhoufuzhi* (Gazetteer of Guangzhou Prefecture), Guangzhou: Yuexiu Shuyuan. 1879; vol.15. <http://read.nlc.cn/OutOpenBook/OpenObjectBook?aid=403&bid=81365.0>

6. Li B. *Sikong Hou Andu Miaoji* (Memorial Temple of Hou Andu, the Minister of Public Works). In Dong G, Ruan Y, Xu S, et al. *Quan tang wen* (Complete Works of Tang Dynasty). Beijing: Zhonghua shuju (Zhonghua Book Company)*.* 1983; vol. 712, p. 7311.

7. Su S. *Chaozhou Han Wengong Miao Bei* (Inscription on the Temple Tablet of Han Wen Gong in Chaozhou). In Kong, FL, ed, *Sushiwenji* (Collected Works of Su Shi). Beijing: Zhonghua Shuju (Zhonghua Book Company)*.* 1986; p509.

18. Li SC. Shuyuanji (Records of the Academy). In Zhou SX, ed, *Chaozhoufuzhi* (Gazetteer of Chaozhou Prefecture). Chaozhou: Baoanzongju*.* 1893; vol 12.

<http://read.nlc.cn/OutOpenBook/OpenTwoObjectBook?aid=403&bid=105381.0&cid=157056>

19. Zhang J. *Qiandao Siming Tujing* (Siming Map and Classics in Qiandao regin), vol. 1. In *Songyuan fangzhi congkan* (Collected Works on Song and Yuan Local Chronicles) Beijing: Zhonghua Shuju (Zhonghua Book Company)*.* 1990; p. 4877.

20. Li T. *Xu Zizhitongjian Changbia* (Continuation of Comprehensive Mirror for Aid in Government: Extended Compilation). Beijing: Zhonghua Shuju (Zhonghua Book Company)*.* 2004; vol. 118, p. 2783.

21. Li GF. *Luoyangmingyuanji* (Famous Gardens in Luoyang). In Chen, Z, Zhang GC, eds. *Zhongguo Lidai Mingyuanji Xuanzhu* (Selected notes of Famous Gadens in China). *Hefei: Anhui kexuejishu chubanshe*. 1983; p. 38

22. Zhu SS. *Lun Songdaiwenhua Zhong de “Meishan Xianxiang”* (On the “Meishan Phenomenon” in Song Culture). Journal of Sichuan University (Social Science Edition), 2004; 03: 105-110.

**Some English references 10-13 are added**.

**The notes in previous reference are moved to corresponding text**:

1) “Xianliang Fanfzheng Zhiyan Jikjian Ke, an examination on Virtuous and Upright Officials Giving Candid Advice.” And “Mishusheng Zhuzuozuolang, Assistant Officer in the Secretariat.” are moved to **Page 8, line 160 to 162** as “Xianliang Fanfzheng Zhiyan Jikjian Ke (an examination on virtuous and upright officials giving candid advice) and was awarded Mishusheng Zhuzuolang (an assistant officer in the secretariat)”.
2) “a region in ancient China, mainly located in the Zhejiang.” is moved to **Page 21, line 433 to 434** as “Mingzhou is in the eastern part of Yue (a region in ancient China, mainly located in the Zhejiang.)”
18. **Question:**

Ethical Considerations:
The manuscript adheres to ethical standards, with clear statements on data availability and no competing interests declared.
**Reply:**

The Declaration of “Data Availability: All relevant data are within the paper and supporting information.” and “Competing interests: The authors declare no conflict of interest.” are resubmitted through submission system.

19. **Question:**

Conclusion:
The inclusion of figures and maps is beneficial; however, augmenting their utility with more detailed captions and in-text explanations would be advantageous. It is important to ensure that each figure is explicitly referenced and thoroughly discussed in the text.
**Reply:**

As we can see from reply of question 16, we check carefully the detailed captions of every Fig. The in-text explanation and reference of the Figs has been checked and modified.

20. **Question:**

Recommendation:
Accept with minor revisions.
To improve their manuscript, the authors should consider addressing the specific points mentioned in order to bolster the clarity, robustness, and overall quality. This will result in a more impactful contribution to our understanding of the cultural and historical landscape of the Song Dynasty.

**Reply:**

In order to improve our manuscript and to make our manuscript to meet the requirements of PLOS ONE, we have made major revision of our manuscript. For instance, we add a new section of “**Research objectives, data sources, data processing, and research method**”. All Figs and Tables are remade to make them clearer. Significant modifications have been made to the supporting information. The original data has been provided in the supporting information. Other detailed correction such as grammar mistakes, smooth transition and so on have been made. According to revision comments, we try our best to answer all of the questions point to point to improve the quality of our manuscript.

**Response to Reviewer #2:**

21. **Question:**

The manuscript is with major flaws, geographical representations of poets does not describe the significance of this research. There should be rationale behind conducting this type of research. Problem statement and research questions should be included. In-text citations and references are in pathetic condition. Methodology is not clear.

**Reply:**

According to this comments, a new section “**Research objectives, data sources, data processing, and research method**” is added after the “**instruction**” section. In this new section, the research objectives, the GIS data collection and processing, and research method are described. Especially, the significance of this research have been described in ‘**Research objectives**’, and the ‘**Research method**’ section describes the methodology used in our research work. All the figures in the revised manuscript have been remade on PLOS ONE's requirements for clarity. The references and citations have also been revised, and some English references are added. In addition, our research work mainly focuses on ancient China, and some Chinese references are inevitably cited, which have no English version. Thus, we try our best to further explain these references in the supporting information.
